# An efficient genetic algorithm for structural RNA pairwise alignment and its application to non-coding RNA discovery in yeast

**DOI:** 10.1186/1471-2105-9-521

**Published:** 2008-12-05

**Authors:** Akito Taneda

**Affiliations:** 1 Graduate School of Science and Technology, Hirosaki University, 3 Bunkyo-cho, Hirosaki, Japan

## Abstract

**Background:**

Aligning RNA sequences with low sequence identity has been a challenging problem since such a computation essentially needs an algorithm with high complexities for taking structural conservation into account. Although many sophisticated algorithms for the purpose have been proposed to date, further improvement in efficiency is necessary to accelerate its large-scale applications including non-coding RNA (ncRNA) discovery.

**Results:**

We developed a new genetic algorithm, Cofolga2, for simultaneously computing pairwise RNA sequence alignment and consensus folding, and benchmarked it using BRAliBase 2.1. The benchmark results showed that our new algorithm is accurate and efficient in both time and memory usage. Then, combining with the originally trained SVM, we applied the new algorithm to novel ncRNA discovery where we compared *S. cerevisiae *genome with six related genomes in a pairwise manner. By focusing our search to the relatively short regions (50 bp to 2,000 bp) sandwiched by conserved sequences, we successfully predict 714 intergenic and 1,311 sense or antisense ncRNA candidates, which were found in the pairwise alignments with stable consensus secondary structure and low sequence identity (≤ 50%). By comparing with the previous predictions, we found that > 92% of the candidates is novel candidates. The estimated rate of false positives in the predicted candidates is 51%. Twenty-five percent of the intergenic candidates has supports for expression in cell, i.e. their genomic positions overlap those of the experimentally determined transcripts in literature. By manual inspection of the results, moreover, we obtained four multiple alignments with low sequence identity which reveal consensus structures shared by three species/sequences.

**Conclusion:**

The present method gives an efficient tool complementary to sequence-alignment-based ncRNA finders.

## Background

The RNA worlds in both experimental and computational fields have recently grown rapidly, and non-coding RNAs (ncRNAs) have increased their importance in life sciences. One of the most important breakthrough from the experimental side is the high-throughput experiments which have unveiled the existence of many non-protein coding transcripts in various species [1,2]. While function-known ncRNAs, which often harbor family-specific conserved secondary structure, such as tRNAs and miRNAs have been intensively studied in detail, no functional annotation has been assigned to a number of known non-protein coding transcripts yet. Since experimental assessment whether all known non-protein coding transcripts are functional or not is quite time-consuming, computational screening for finding the ncRNAs with conserved secondary structure is an important step for determining not only expressed but also functional transcripts. Computational comparative genomics is a powerful approach to identify ncRNA candidates with conserved secondary structure from genomic sequences. To date, sequence-alignment-based ncRNA finders such as RNAz [3], QRNA [4] and EvoFold [5] have been successfully applied to ncRNA discoveries from various complete genomes [6-10]. While these methods are so efficient that they can be applied to genome-scale analysis, sequence-alignment-based methods need a pre-computed alignment as an input data. In other words, they implicitly assume that an adequately accurate RNA sequence alignment can be obtained by using pure sequence alignment method (e.g. ClustalW) which does not explicitly consider conserved secondary structure. Although this assumption is acceptable for the RNA sequences with relatively high sequence identity, sequence-alignment-based methods can fail to indentify the ncRNAs with low sequence identity; this is because conserved secondary structure should be taken into account to accurately align structured RNA sequences which are poorly conserved at sequence level.

Finding related structured RNA sequences with low sequence identity from genomic sequences is more challenging compared to the case of high sequence identity. This is mainly due to the high computational complexities of structural RNA sequence alignment algorithms which explicitly take secondary structure into account (in the present paper, the term "structural RNA sequence alignment" is used to indicate "simultaneously determining RNA sequence alignment and conserved secondary structure without pre-defined secondary structure annotation"). For example, the computational complexities of Sankoff's algorithm which is the most basic algorithm for structural RNA sequence alignment are *O*(*N*^3*M*^) in time and *O*(*N*^2*M*^) in space, where *N *and *M *are the length and the number of RNA sequences to be aligned, respectively [11]. Even when one performs pairwise alignment, Sankoff's algorithm needs *O*(*N*^6^) in time and *O*(*N*^4^) in space. To improve the computational speed and memory usage of structural RNA sequence alignment, various variations of Sankoff's algorithm have been intensively studied [12-21].

So far, Dynalign [13] and Foldalign [14] which are variations of Sankoff's algorithm, have been applied to the pairwise comparative genomics for novel ncRNA discoveries[22,23]. Indeed these 'structure-based' ncRNA finders have successfully predicted a number of ncRNA candidates with low sequence identities, these calculations needed long computational times and large computational resources. Although these programs have been updated recently and the latest versions are faster compared to their older versions, it is still time consuming to apply these programs to genome-scale applications. Recently an efficient structural RNA sequence alignment algorithm, LocARNA, has been proposed[15]. To our knowledge, however, there is no report related to the ncRNA discovery by using LocARNA. Since genomic scans by previous structural RNA sequence alignment methods are time consuming and need large computational resources in general, further development of efficient and accurate structural RNA sequence alignment algorithm is important to accelerate the genome-scale prediction of the ncRNAs with low sequence identities. Recently, CMfinder, which is structural RNA sequence alignment algorithm not for pairwise but for multiple RNA sequence alignment, has successfully predicted a number of novel structured RNA motifs from the ENCODE regions with low sequence identities [24].

In the present paper, we propose an improved genetic algorithm (GA), Cofolga2, for structural RNA pairwise alignment which uses the base pairing probabilities (BPPs) by RNAfold[25] to evaluate the structural term of the objective function instead of directly using the free energy parameters as its version 1 does [21]. Since the present algorithm is efficient in both time and memory usage, we applied the algorithm to the pairwise comparisons between eukaryotic complete genomes to search for novel ncRNA candidates from low sequence identity regions. The rest of the present paper is organized as follows. First we describe the present structural RNA sequence alignment algorithm and a strategy for our comparative genomics in the 'Methods' section. In the next section, we show the results of the benchmark and the comparison between the present algorithm and previous ones, discussing the performance of our alignment algorithm. Finally, we present the detail of the ncRNA candidates obtained by the pairwise genome comparisons between *S. cerevisiae *and other six fungi.

## Methods

In Cofolga2 algorithm, we employ a GA to search for the optimal solution of structural RNA pairwise alignment. Cofolga2 is an updated version of the previously proposed GA [21] which performs structural RNA pairwise alignment based on minimization of free energy and the GA frameworks proposed in RAGA [26] (in the present paper, we call the previous version as Cofolga1). Cofolga2 runs much faster compared to Cofolga1; this is mainly due to the improvement in the formulation of objective function and introduction of a new technique for random alignment generation. In standard GA, various GA operators (crossovers and mutations) are iteratively applied to a population of individuals (solutions) to search for the optimal solution with the highest value of a given objective function (OF) [27]. In the Cofolga algorithms, an individual of GA is represented by a pairwise alignment. This is because structural RNA sequence alignment problem can be decomposed into sequence alignment and alignment folding, and the optimal alignment folding is uniquely defined for a given alignment. As a result, the conformational space to be explored in the present structural RNA pairwise alignment is reduced to that of non-structural pairwise sequence alignment.

The OF of Cofolga2 is represented by the following formula:

(1) *f *= *s *+ *wP*,

where *s *is a sequence alignment score, *P *is a term for consensus secondary structure; *w *is a parameter for controlling the weights of *s *and *P*.

For a given pairwise alignment of RNA sequence A and B, the *P *in Equation 1 is evaluated as follows.

First an averaged BPP matrix ***B ***is constructed:

(2) $$b_{ij}=\{\begin{array}{ll} (p_{k_{i}l_{j}}^{\text{A}}+p_{m_{i}n_{j}}^{\text{B}})/2 & \begin{array}{cc} \begin{array}{cc} p_{k_{i}l_{j}}^{\text{A}}\neq 0 & \text{and} \end{array} & p_{m_{i}n_{j}}^{\text{B}}\neq 0 \end{array}\\ 0 & \text{otherwise}. \end{array}$$

In Equation 2, *b*_*ij *_is the matrix element of ***B***, where *i *and *j *indicate the column positions in the pairwise alignment; *k*_*i *_and *l*_*j *_(*m*_*i *_and *n*_*j*_) are the nucleotide positions in sequence A (sequence B) corresponding to column position *i *and *j *in the pairwise alignment, respectively. The BPPs of single sequence A and B, $p_{kl}^{\text{A}}$ and $p_{mn}^{\text{B}}$, are computed by RNAfold [25]. Secondly, the *P *is evaluated by taking a summation of the elements in matrix ***B***:

(3) $$P=\sum_{i<j}b_{ij}.$$

It is noteworthy that Equation 3 can be applied to any type of pseudoknotted structure without modification. This means that once the BPP matrixes taking pseudoknots into account are given, Cofolga2 algorithm can perform structural RNA sequence alignment of pseudoknotted RNAs without an increase of computational costs compared to the case of non-pseudoknotted RNAs.

**Figure 1:**
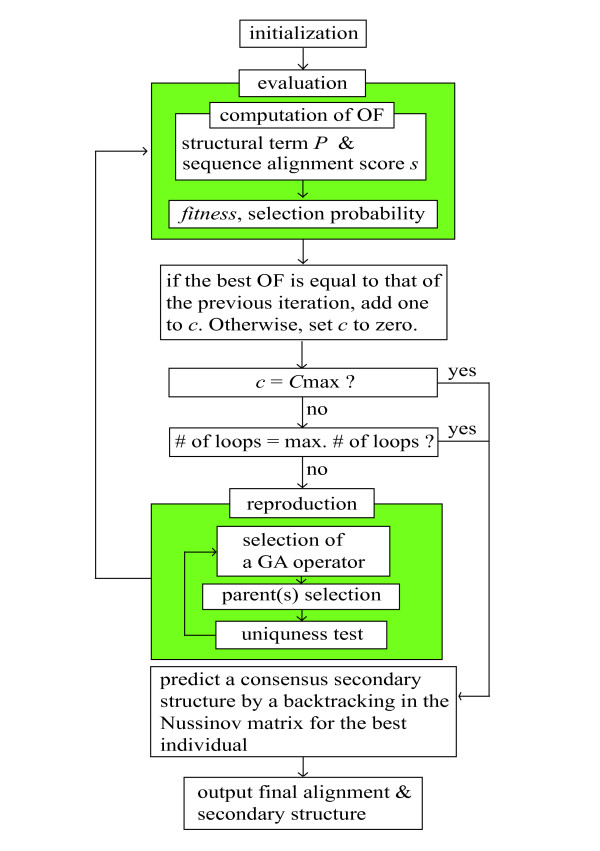
**Schematic flowchart of Cofolga2 algorithm**. Cofolga2 algorithm is composed of three GA steps (initialization, evaluation, and reproduction) and a postprocessing step. In initialization, a population of individuals is randomly generated by weighted stochastic backtracking. In evaluation step, the objective function of each individual is evaluated and then fitness and selection probability are assigned to each individual. In reproduction, half of the population is replaced by new individuals to update the population. The iteration between evaluation and reproduction stops when one of the following conditions is satisfied: the best OF is not updated continuous *C*_max _times, or the number of iteration reaches a pre-defined maximum iteration number. The *C*_max _and the maximum iteration number are parameters given by user. A consensus secondary structure prediction for the optimal alignment is performed as a postprocessing, where the Nussinov matrix constructed by averaged base pairing probabilities is backtracked.

The flowchart of Cofolga2 algorithm is shown in Figure 1. In accordance with the standard GA, first, initialization is done to randomly generate an initial population, and then evaluation and reproduction procedures are iteratively performed to update the population. This iteration stops when the number of iterations reaches a user-defined maximum number or when no improvement has been observed for a user-defined number of iterations.

As mentioned above, Cofolga2 was developed based on Cofolga1. In the following subsections, we will focus on explaining the detail of algorithms newly introduced for Cofolga2. Algorithmically common parts between the two versions will be briefly explained.

### Initialization

An initial population of solutions is generated by adding a randomly generated pairwise alignment to the population one by one until the number of individuals reaches a user-defined population size. The random pairwise alignments are computed by using weighted stochastic backtracking (for detail, see next subsection). In weighted stochastic backtracking, the randomness of the alignment can be controlled with a '*noise*' parameter where larger *noise *gives more randomized alignment; based on our experience, we used *noise *= 0.1 – 0.4 to obtain random alignments.

In addition to the random alignments, a non-random alignment taking structure information into account (computed by weighted stochastic backtracking with a very small noise such as *noise *= 1.0 × 10^-4^) and a non-structural Needleman-Wunsch alignment [28] can also be included in the initial population through a command line option (a '-nrd' option). When invoked with the '-nrd' option, Cofolga2 works as a refinement program which improves the two non-random alignments. Inclusion of the non-structural alignment improves the quality of the alignments with a relatively high sequence identity. In the default setting of Cofolga2, duplicated individuals in one population are not allowed throughout the run.

### Weighted stochastic backtracking

In the initialization step of the alignment algorithms utilizing GA such as SAGA [29] and RAGA [26], it is necessary to generate a number of random alignments. For example, in RAGA algorithm, random pairwise alignments are computed by using a Dynamic Programming with Added Noise (DPAN) in which random alignments are obtained by adding small random noises to each DP matrix elements [30]. Since DPAN constructs a DP matrix in accordance with non-structural Needleman-Wunsch algorithm, structural information is completely lacked in such a calculation. To obtain a better initial guess for the structural RNA sequence alignment, structural information should be taken into account.

To generate random pairwise alignments which reflect structural information, we developed weighted stochastic backtracking. In this algorithm, first, we construct the DP matrix for a pairwise alignment according to StrAl algorithm [31]. StrAl algorithm is an efficient structural alignment algorithm, and it was derived from an affine gap version of Needleman-Wunsch algorithm [28]. An essential difference between StrAl and the Needleman-Wunsch algorithm is their similarity scoring scheme. In StrAl algorithm, the following similarity score *s*_*ij *_is used when constructing the DP matrix for pairwise alignment instead of the nucleotide substitution matrix *d *(*A*_*i*_, *B*_*j*_) alone:

(4) $$s_{ij}=\alpha \left(\sqrt{\kappa_{i}^{\text{A}}\kappa_{j}^{\text{B}}}+\sqrt{\lambda_{i}^{\text{A}}\lambda_{j}^{\text{B}}}\right)+d(A_{i},B_{j})\sqrt{\mu_{i}^{\text{A}}\mu_{j}^{\text{B}}}$$

(5) $$\kappa_{i}^{\text{X}}=\sum_{k<i}p_{ik}^{\text{X}},\lambda_{i}^{\text{X}}=\sum_{k>i}p_{ik}^{\text{X}},$$

(6) $$\mu_{i}^{\text{X}}=1-\left(\kappa_{i}^{\text{X}}+\lambda_{i}^{\text{X}}\right)$$

where *s*_*ij *_indicates the similarity score between position *i *of sequence A and position *j *of sequence B, and *α *is the ratio of structure over sequence similarity. Nucleotide substitution matrix element *d*(*A*_*i*_, *B*_*j*_) is the substitution score between the *i*th nucleotide of sequence A and the *j*th nucleotide of sequence B. In the present study, we used RIBOSUM85-60 [32] for *d*(*A*_*i*_, *B*_*j*_) and *α *= 0.9 which was taken from the StrAl paper [31]. Base paring probability vectors $\kappa_{i}^{\text{X}}$, $\lambda_{i}^{\text{X}}$, and $\mu_{i}^{\text{X}}$ are the probabilities defined for the position *i *of sequence X (= A or B) which represent probabilities of being paired upstream, paired downstream, and unpaired, respectively. The affine gap penalties which we used for weighted stochastic backtracking are also taken from the StrAl paper [31].

After the construction of the DP matrix, we backtrack the DP matrix in accordance with a roulette wheel selection. Roulette wheel selection is a selection method frequently used in GAs, in which one of all choices is randomly chosen in accordance with the probability proportional to the size of a virtual 'slot' assigned to the choice.

The size of the slots is determined by the following scaling function:

(7) $$slot_{i}=\frac{noise}{h_{\max}-h_{i}+noise},$$

where index *i *corresponds to a backtracking path at a node of the DP matrix (*i *= 1, 2, 3 for pairwise alignment), *h*_*i *_is the score difference between the current node and the neighboring node for path *i*, and *h*_max _is the largest *h*_*i *_among *h*_1_, *h*_2_, and *h*_3_. Larger noise parameter *noise *generates a more randomized alignment. A backtracking path is chosen in accordance with backtracking probability π_*i *_which is defined as follows:

(8) $$\pi_{i}=slot_{i}/\sum_{i=1,2,3}slot_{i}$$

While backtracking, a real random number ranging from 0 to 1 is generated at each node and used to select a next path to be backtracked.

**Figure 2:**
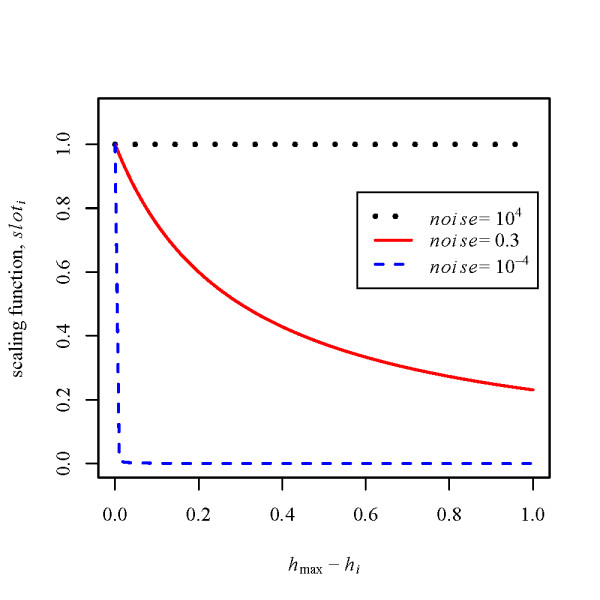
**Scaling function for weighted stochastic backtracking**. The curves by the proposed scaling function, *slot*_*i *_= *noise*/(*x *+ *noise*), are plotted, where *x *is the score difference between the optimal and the other backtracking path (i.e. *x *= *h*_max _- *h*_*i *_in Equation 7). In the figure, dotted line, solid line, and dashed line indicate the curves for *noise *= 10^4^, *noise *= 0.3, and *noise *= 10^-4^, respectively (*noise *= 10^4 ^and *noise *= 10^-4 ^are drawn as examples of two extreme cases). For any *noise *> 0, the optimal path (*x *= 0) has *slot*_max _= 1, while damped slot sizes (< 1) are assigned to the non-optimal DP paths (*x *> 0).

In Figure 2, the curves drawn by the scaling function are plotted. As can be seen from the figure, higher *noise *increases the probability to choose low scoring paths, while *noise *→ 0 means the optimal alignment. Thus, the randomness of weighted stochastic backtracking is controllable through the single parameter *noise*. It is noteworthy that the principles of weighted stochastic backtracking can easily be applied to any type of DP algorithm, e.g. those of Nussinov's algorithm [33] and Sankoff's algorithm [11].

### Evaluation

The OF, *f *in Equation 1, of each individual is evaluated in this step, where the alignment score *s *is calculated by using the RIBOSUM85-60 [32]. Opening and elongation gap penalties are left as free parameters. After the evaluation of the OF, the fitness of each individual is computed from the OF as *fitness *= *OF *- (the lowest *OF *in the population), and then a selection probability proportional to the fitness is calculated for each individual. The selection probability is used in reproduction step as the size of virtual slots for the roulette-wheel selection of parent individuals.

### Reproduction

**Figure 3:**
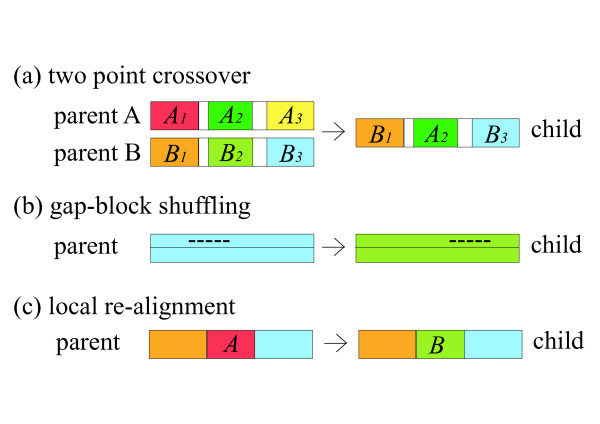
**Schematic illustration of GA operators**. Different colors of the blocks indicate different alignment blocks. White blocks indicate equivalent blocks between two pairwise alignments. (a) *Two-point crossover *creates a child alignment by concatenating at most three alignment blocks separated by equivalent blocks. When the number of the separated blocks is larger than three, the smallest blocks are merged to neighboring blocks in an iterative manner. (b) *Gap-block shuffling *shifts a randomly selected continuous gap in a random direction. (c) *Local re-alignment *re-aligns a randomly selected small alignment region. In this example, the central region of the alignment is modified, while the flanking regions are not changed.

In reproduction step, half of the population with the lowest OFs is replaced by new child individuals. The child individuals are generated by applying GA operators to the parent individuals randomly selected from the population. We use a modified set of the GA operators taken from Cofolga1[21], which is comprised of two crossovers (random and greedy two-point crossovers) and three mutations (random and greedy gap-block shuffling operators, local re-alignment with weighted stochastic backtracking). Each GA operator is invoked with an equal probability and applied to one or two randomly selected parent individual(s); the crossovers need two parents, while the other operators are applied to a single parent. Selection of parent(s) is performed by roulette-wheel selection where the selection probability of each individual is used as the size of the slots. The GA operators are schematically illustrated in Figure 3. Crossover operators construct a new alignment by concatenating 'alignment blocks' taken from two parent individuals. Gap-block shuffling operator 'shuffles' a gap block (a block of continuous gaps) by a random shift size in a random direction. The maximum size of the gap shift is defined by shift size parameter *max*_*shift*.

In 'local re-alignment with weighted stochastic backtracking', a randomly selected small region of the alignment is re-aligned using weighted stochastic backtracking. The region to be re-aligned is selected by the following gap-sensitive procedure. First, we initialize *ρ*[*i*] = 1 for all *i*, where *i *indicates the column position of the alignment. Secondly, we scan the alignment with a sliding window of *W *columns. While scanning the alignment, we count the number of gaps in each window and add the number to the *ρ*[*i*] whose *i *is the center of the sliding window. Thirdly, a column position *k *is randomly selected in accordance with the probability proportional to *ρ*[*i*]. Finally, we define the region to be re-aligned around the *k*. The width of the region is randomly determined between *l*_min _and *l*_max_. When we meet a trivial case (i.e. when one of the alignment rows included in the region has no nucleotides), this operator is rejected and a next GA operator is randomly invoked. In this procedure, the *W*, *l*_min _and *l*_max _are the parameters to be given by user.

In general, GA has several free parameters such as population size and iteration number which have to be given before execution. To reduce the number of such free parameters, we introduced unification parameter *L*. This *L *defines several parameters for the GA operators simultaneously through the following relationships: *max*_*shift *= *l*_max _= *L *and *l*_min _= (*W *- 1)/2 = ⌊*L*/2⌋. Hence once *L *is given, *max*_*shift*, *l*_max_, *l*_min_, and *W *are determined and only *L *is left as a free parameter. Unification parameter *L *controls the degree of modification, i.e. larger *L *leads to a wider search in the conformational space by the mutation operators. It is noted that too large *L *can cause a slow convergence of the GA.

In the nomenclature of the GA operators, a 'greedy' means that the operator increases the OF of the child individual compared to that of its parents. Cofolga2 uses 'greedy' operators while Cofolga1 uses 'semi-greedy' operators. The 'greedy' operators of Cofolga2 reject the child individuals which do not satisfy (the OF of the child individual) > (the OF of the parent individual(s)) while the 'semi-greedy' operators of Cofolga1 does not. Cofolga2 does not utilize 'anchor point for mutation operators' which is used in Cofolga1 to avoid gap insertion into highly conserved regions of an alignment (subsection 2.3.2 in [21]). Local Cofolga operator (*ibid*., subsection 2.3.6) is also not used in Cofolga2.

### Consensus structure prediction by a postprocessing

Cofolga2 predicts the consensus secondary structure for the final alignment by backtracking in the averaged BPP matrix as a postprocessing of the GA procedures. To more accurately predict a consensus secondary structure based on the alignment computed by Cofolga2, it is better to use an alignment folding program such as RNAalifold [34] or Pfold [35] as a postprocessing.

### Measures for assessing alignment quality

The quality of pairwise alignments was assessed with structure conservation index (SCI) and sum-of-pairs score (SPS). SCI and SPS were evaluated by RNAz [3] and bali_score.c [36], respectively.

### Determination of the free parameters

In addition to the population size and maximum iteration number of the GA, Cofolga2 has six free parameters: *C*_max_, *noise*, *w*, gap opening and elongation penalties for *s*, and *L *. We optimized these six parameters with fourteen pairwise alignments taken from the k2 dataset of BRAliBase 2.1 [37]. The training RNA sequences are tRNAs, 5S rRNAs, and SRP RNAs with high or low sequence identities and high or low SCIs. For tRNA, alignments with a moderate sequence identity were also used. The file names of the RNA sequences are listed in Additional File 1. The parameter space to be explored was represented by a coarse grid and the parameter set corresponding to the grid point which scored the highest (mean SPS) × (mean SCI) was adopted as the optimal parameter set (*C*_max _= 50, *noise *= 0.3, *w *= 50, gap opening = 30, gap elongation = 4, and *L *= 50). Throughout this optimization, we used a population size and maximum iteration number fixed to relatively large values, 150 and 150, respectively. The results presented at the Results and discussion section were obtained with this optimal parameter set. The population size and maximum iteration number are left as free parameters.

### Benchmark and comparison of alignment quality

We performed an alignment quality benchmark using BRAliBase 2.1 [37] from which RNA sequence pairs and their reference alignments were taken. In addition, we performed performance comparison with other structural and non-structural sequence alignment programs using the benchmark. In the performance comparison, we compared Cofolga2 with five structural and three non-structural alignment methods. The programs and command line options are summarized in Table 1. To perform the comparisons on an equal footing, global alignment mode was used for local alignment programs, Foldalign and LocARNA.

**Table 1:** Alignment programs used for performance comparisons

program	version	command	structural?	reference
Foldalign	2.1.0	*foldalign-global seq _filename*	yes	[48]
LocARNA	0.99	*mlocarna-p-sequ-local = 0 seq _filename*	yes	[15]
Dynalign	*	*cat opt_ file *| *dynalign*	yes	[13]
LaRA	1.3.1	*lara-i seq_ filename*	yes	[55]
StrAl	0.5.2	*stral seq _filename*	yes	[56]
MAFFT	6.240	*ginsi seq _filename*	no	[57]
ClustalW	1.83	*clustalw seq_ filename-outfile = out_ filename*	no	[58]

In this table, 'program' and 'version' columns indicate program names and their versions, respectively. In 'command' column, executable file names and options we used are listed. In 'structural?' column, 'yes' indicates structural RNA sequence alignment program, and 'no' represents non-structural sequence alignment program. RNAz 1.0 with a default setting was applied to the alignments provided by MAFFT and ClustalW. (*) Dynalign version 4.5 (with *M *= -99) was used for the alignment quality benchmark, and Dynalign version 4.3 (with *M *= 8) was used for the ncRNA prediction benchmark, where we used the values recommended by Dynalign for the other parameters except for the maximum number of structures = 1. The parameters and file names were written in *opt_file *and used to run Dynalign.

### Benchmark for the sequence pairs with low identities

In addition to the BRaliBase 2.1 benchmark, we have performed a benchmark with the sequences which have identities ≤ 40% and lengths of 100 to 150 nt. The sequences were extracted from the internal transcribed spacer 2 (ITS2) database [38], where the sequences and annotated structures of *Stramenopiles *and "the original 5,000 sequences and structures" (ITS2.html, [39]) were used. Sequence identities were measured after aligning two ITS2 sequences using MAFFT (see Table 1). We have performed non-redundant processing with a cutoff of 90%id. As a result, we obtained twenty-five ITS2 sequence pairs (the ITS2 dataset can be browsed at the Cofolga2 website [40]); the average sequence identity of the dataset is 33%. This benchmark was performed for Cofolga2, Foldalign 2.1.0, and LocaRNA. Since annotated secondary structures are given in the ITS2 database and reference alignments are not provided, the prediction accuracy for this benchmark was measured based on how correctly annotated (reference) base pairs are predicted. The correctness of the predicted base pairs was assessed with the approximated Matthews correlation coefficient (Equation 5 in [41]), *CC*, proposed by Gorodkin *et al*..

### SVM classification between true ncRNAs and shuffled data

To predict ncRNAs on the basis of the pairwise alignment computed by Cofolga2, we trained SVM by using a SVM package software, LIVSVM (version 2.84) [42]. The elements of the feature vector for the SVM are as follows: OF, alignment length, and A, C, and U frequencies of the two sequences. These quantities except for the OF were calculated after eliminating all gapped columns of the alignment. The alignments < 50 nt were removed from the input before SVM processing. This format of the feature vector is taken from the paper describing the ncRNA finding by Dynalign [43]. We use a default kernel (radial basis function kernel), and the prediction result of the SVM is outputted as a classification probability. To construct positive training and test datasets, we extracted 5,010 pairwise alignments from the k2 dataset of BRAliBase2.1 [37]. The sequence identity of this dataset ranges from 16% to 75% and the dataset comprises thirtytwo RNA families. This original dataset was divided into two sub-datasets in a ratio of 1:2 (1,670 alignments for training, 3,340 alignments for test). Negative data were generated by removing all gapped columns of the positive alignments and shuffling the gap-free alignments. Two negative alignments were generated for each positive alignment, consequently we obtained 3,340 negatives for training and 6,680 negatives for test. The shuffling was performed by shuffle-aln.pl [34] with a '-m complete' option. After the training, we obtained a test accuracy of 87.7%.

### Visualization of ncRNA prediction performance

When the performance of prediction methods depends on their own cutoff value, comparison of the methods becomes not straight forward, since varying the cutoff value leads to a simultaneous change of sensitivity and specificity (i.e. there is a tradeoff between sensitivity and specificity).

In the present study, we used receiver operating characteristic (ROC) curve for visualizing the tradeoff between sensitivity and specificity for a range of cutoff value. The ROC curve has been used by Uzilov *et al*. to compare the performance of ncRNA finders [43].

ROC curve is defined as *sensitivity *vs *false positive rate *plot; *sensitivity *and *false positive rate *are defined as follows:

(9) $$\begin{array}{c} false\;positive\;rate=(1-specificity)=\frac{FP}{TN+FP},\\ \begin{array}{cc} sensitivity=\frac{TP}{TP+FN}, & specificity=\frac{TN}{TN+FP}, \end{array} \end{array}$$

where, *TP*, *FP*, *TN *and *FN *are the number of true positives, false positives, true negatives and false negatives, respectively. In the case of comparative ncRNA prediction, *sensitivity *indicates how many positive alignments (i.e. alignments containing true ncRNAs) are correctly predicted as ncRNA; *false positive rate *represents how many negative alignments are misclassified as ncRNA. For example, *false positive rate *= 1% means that one false positive is found when we evaluate 100 negative alignments.

### Genome sequences

The genome sequences (excluding mitcondorial chromosome) of *S. cerevisiae *and the contigs of other six fungi (*S. bayanus*, *S. castellii*, *S. kluyveri*, *S. kudriavzevii*, *S. mikatae*, and *S. paradoxus*) were downloaded at *Saccharomyces *Genome Database (SGD) [44]. Annotated fasta files for *S. cerevisiae *(orf_coding.fasta, rna _coding.fasta, NotFeature.fasta, and other_features_genomic.fasta) were also downloaded at SGD. We masked the genome sequences of *S. cerevisiae *according to the other_features_genomic.fasta file to remove repetitive sequences from the genome sequences.

### Pairwise comparison of genomic sequences

**Figure 4:**
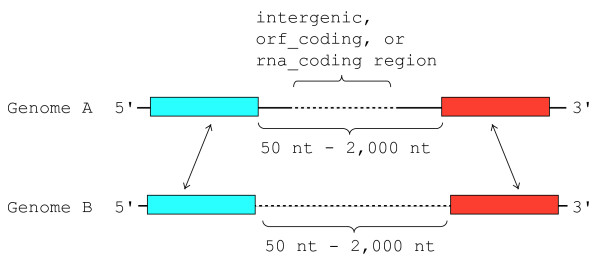
**Definition of the target region to be scanned by Cofolga2**. The conserved sequences are indicated by boxes, and matching sequences are represented by arrows. In this figure, Genome A conrresponds to *S. cerevisiae*, and Genome B corresponds to an other fungal genome. Dashed lines indicate the sequences belonging to the target region. A situation such that the arrows are crossing each other is not allowed. If a part of intergenic, orf_coding, or rna_coding region overlaps the conserved region, the overlapped part is excluded from the target region.

To efficiently search for ncRNA candidates with low sequence identity, we focused on our scan to the relatively short (50 bp to 2,000 bp) low-identity regions located between two regions which are conserved at sequence level. By exploring the regions neighboring such conserved regions, we can expect to find the ncRNAs hidden in a conserved synteny. The conserved regions were detected by using WU-BLAST [45] comparison (cutoff E-value = 10^-3^) between *S. cerevisiae *and the other fugal genomes. Then we constructed 'target regions', which are the regions scanned by Cofolga2, as follows. First, the *S. cerevisiae *genome sequence was divided into intergenic (NotFeature), orf_coding, and rna_ coding sequences in accordance with the annotations in SGD [44]. Then target region was defined for each divided *S. cerevisiae *sequence as illustrated in Figure 4 if the divided sequence overlaps the low-identity region located between the conserved regions. As a result, the target regions which we obtained by the WU-BLAST comparison cover 2,196,982 bp of the *S. cerevisiae *genome (this corresponds to 18% of all auto chromosomes of *S. cerevisiae*).

The present approach for generating the target regions is similar to that used in the genome comparison between human and mouse by Torarinsson *et al*. [46]. Compared to their approach, however, ours is more conservative since it requires the target regions to be sandwiched by two conserved regions, while Torarinsson *et al*. scanned the regions neighboring to singly conserved regions. In other words, our definition is a subset of that of Torarinsson *et al*..

We scanned each target region using a dual sliding window according to the following procedure. Let us call the two genome sequences belonging to a target region genome A and genome B. Subsequences were generated by moving a sliding window of 150 nt with a shift size of 50 nt on each genome sequence, and then all-vs-all pairwise alignment between the subsequences of genome A and those of genome B was performed with Cofolga2. After the comparison, each pairwise alignment was processed by the trained SVM to assign a SVM classification probability to discriminate whether the pairwise alignment contains ncRNA candidates or not.

## Results and discussion

### Convergence test with respect to GA population size and iteration number

**Figure 5:**
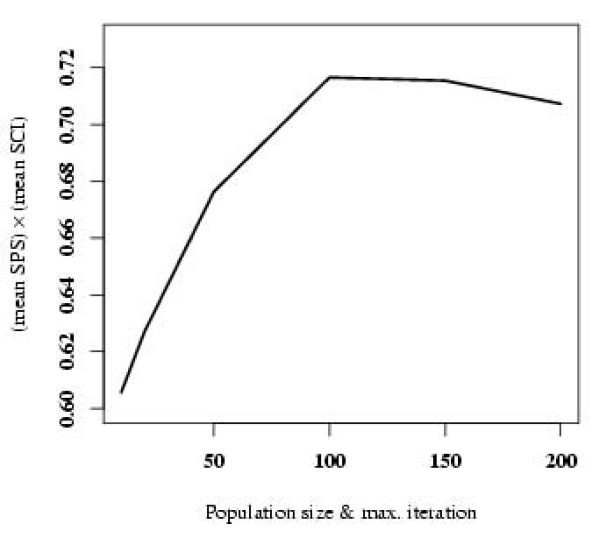
**Convergence property with respect to GA population size and iteration number**. This figure shows (mean SPS) × (mean SCI) as a function of GA population size (= iteration number). The means were taken over five runs with different initial random numbers.

To know how the GA population size and iteration number affect the alignment quality, we studied the population size and iteration number dependence of the Cofolga2's performance, where we define population size = iteration number to reduce the number of free parameters. Figure 5 shows the (mean SPS) × (mean SCI) for the fourteen sequence pairs in Additional File 1 as a function of population size. As can be seen from the figure, the (mean SPS) × (mean SCI) is almost saturated between population size 50 and 100. Based on this observation, we used population size (= iteration number) = 50 for the benchmarks and ncRNA discovery in the present study.

### RNA alignment benchmark and performance comparison with other methods

**Figure 6:**
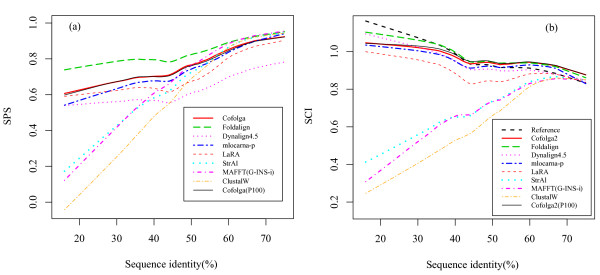
**RNA alignment benchmark with the k2 dataset of BRAliBase 2.1**. Alignment quality benchmark results for (a) sum-of-pair score (SPS) and (b) structure conservation index (SCI). The results for Cofolga2 with a population size = 50 and 100 are denoted by 'Cofolga' and 'Cofolga(P100)', respectively. The curves were drawn by lowess smoothing with a smoother span of 0.3.

Figure 6 shows the benchmark results for Cofogla2 and other programs for structural or non-structural sequence alignment. In this benchmark, 5,010 pairwise alignments (≤ 75%id) taken from the k2 dataset of BRAliBase 2.1 are used. The programs used for the comparison are summarized in Table 1. In this performance test, as can be seen from Figure 6, Cofolga2 outperformed the light-weighted programs (StrAl, LaRA, and LocARNA, and the non-structural alignment programs) at ≤ 50%id in both SPS and SCI. In addition, Cofolga2 showed a performance comparable with the other structural RNA alignment programs in SCI and was the second-best method between 30%id and 50%id in SPS, where Foldalign revealed the best performance. When the fourteen training sequence pairs were excluded from the dataset, the identical conclusion was obtained.

Since GA is a sampling method utilizing random number, it is important to know how an initial random number affects the alignment quality. To examine random number dependence of Cofolga2, we performed five independent runs for the k2 dataset with different initial random numbers. As a result, we confirmed that the differences between the benchmark results due to the difference in initial random number are very small for a wide range of sequence identity (Additional File 2).

In the benchmark with the ITS2 dataset, we found that Cofolga2 showed the best performance (the averaged *CC*s for Cofolga2, Foldalign 2.1.0, and LocaRNA are 0.42, 0.30, and 0.38, respectively).

### Computational time and memory usage

**Figure 7:**
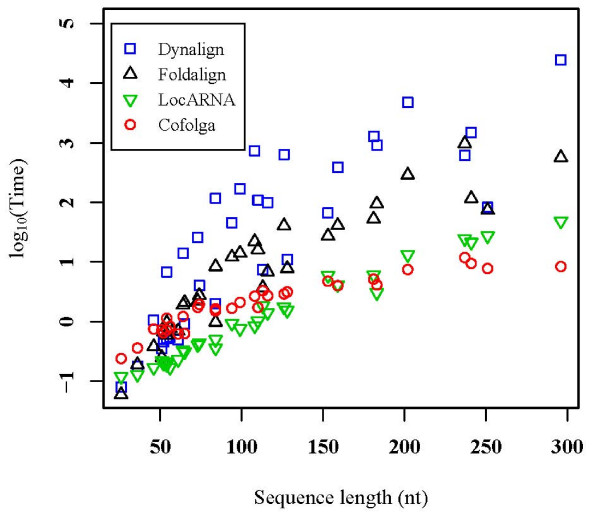
**Comparison of computational times**. Each symbol corresponds to the averaged computational time and averaged sequence length of one RNA family, where time is represented as log_10_(time in seconds).

The computational times (including those for the BPP computation by RNAfold) measured for Cofolga2 and other structural RNA sequence alignment methods are shown in Figure 7. The computational times were measured with a Xeon PC (2.4 GHz/3 GB RAM/Red Hat Linux 9.0).

For the RNA families shorter than approximately 85 nt, Foldalign showed computational times comparable with Cofolga2. For longer RNA families, Foldalign was much slower than Cofolga2 except for K_ chan_ RES (the data of K_ chan_ RES can be found at 113 nt in Figure 7). The computational times of Dynalign were in general much longer than the other methods in the present benchmark. In addition, the computational times of Foldalign and Dynalign were not scaled monotonically with respect to sequence length. This could be due to the pruning algorithm of Foldalign and the constraint used in Dynalign, i.e. when these accelerators do not work well the programs become slower.

LocARNA was faster than Cofolga2 up to approximately 150 nt. For longer RNA families, however, the computational time of LocARNA became comparable with or longer than that of Cofolga2, e.g. average computational times of Cofolga2 and LocARNA for Cobalamin (202 nt) were 7.5 sec. and 13.2 sec., respectively.

To examine the memory usage of Cofolga2, we performed a structural alignment of two SRP _euk _arch RNAs, AP003253.3 (317 nt) and AC005275.1 (305 nt) taken from the BRAliBase 2.1 k2-dataset (the file name of the sequence pair is included in Additional File 1). Since this computation is one of the largest calculations in the present study, we can estimate the upper bound of the memory usage from the result. Consequently, we found that Cofolga2 needs only 10.0 MB RAM to perform the calculation. This memory usage is smaller than or comparable with those of other latest structural RNA sequence alignment methods. According to literature [47], Foldalign 2.1.0, Dynalign 4.5, and LocARNA 0.99 need at least 17.3, 13.3, and 7.6 MB RAM, respectively, to align the 5S rRNAs with an average sequence length of 119.4 nucleotides.

### SVM training results and ncRNA prediction benchmark

**Figure 8:**
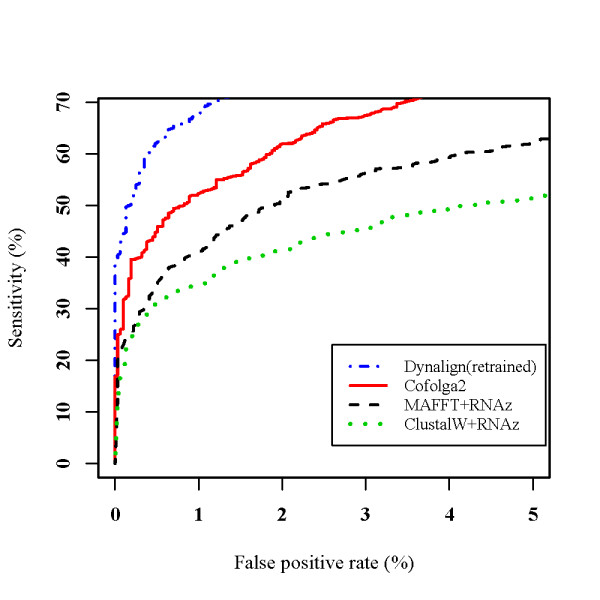
**Comparison of ncRNA prediction performance**. The ROC curves drawn for the alignments ≤ 50%id. The ROC curve obtained using Dynalign 4.3 with the SVM model re-trained in the present study is denoted by 'Dynalign(retrained)'.

In Figure 8, ROC curves by Cofolga2, RNAz, and Dynalign are plotted. To make the plot, first we ran each alignment program for the SVM test data, and then extracted the pairwise alignments satisfying (alignment length after removing gapped columns) ≥ 50 nt and %id ≤ 50%, where the sequence identity based on the BRAliBase 2.1 alignments was used. As a consequence, each ROC curve was drawn based on approximately 4,700 RNA alignments. In Figure 8, "ClustalW+RNAz" indicates that an alignment is constructed by ClustalW and then the alignment is evaluated by RNAz to predict whether the alignment contains ncRNA candidates or not. We used ClustalW and MAFFT to construct input pairwise alignments for RNAz, since ClustalW is the standard sequence alignment program and MAFFT is the best non-structural sequence alignment method in accordance with the previous benchmark performed with BRAliBase 2.1 [37]. In our comparison, we ran Dynalign 4.3 (maximum separation parameter *M *= 8 was used) not with the original SVM model trained in [43], but with a SVM model which was re-trained with the training dataset for the SVM model of Cofolga2. This is because the original SVM model of Dynalign was trained with only tRNA and 5S rRNA sequences, and the Dynalign with the original SVM model showed a poor prediction performance (data not shown) in our benchmark where more RNA families are included. In addition, we did not include Foldalign in the ncRNA prediction benchmark using BRAliBase 2.1, since the ncRNA prediction by Foldalign needs flanking sequences of a ncRNA sequence to obtain a statistical value [48], and the ncRNA sequences of BRAliBase 2.1 do not have flanking sequences. As can be seen from Figure 8, Cofolga2 outperformed RNAz when sequence identity is lower than 50%. Although the re-trained Dynalign showed better prediction results compared to Cofolga2, Dynalign was much slower compared to Cofolga2.

When one performs a genomic scan, it is important to use a cutoff value which gives a very low false positive rate, since genome-scale calculations usually process a number of sliding windows containing negative data. To reduce the false positive rate as small as possible, we chose a cutoff *P*_*SVM *_= 0.9 whose *sensitivity *and *false positive rate *are 25.3% and 0.06%, respectively. Cofogla2 with this cutoff *P*_*SVM *_gives a better false positive rate compared to RNAz (e.g. the false positive rate of MAFFT+RNAz was 2.9 times larger than that of Cofolga2 at *sensitivity *= 27.5%).

### Comparative prediction of yeast ncRNAs

We obtained 6,349 target regions whose average sequence lengths for *S. cerevisiae *and the other fungi are 446 bp and 980 bp, respectively. These target regions cover 2,196,982 bp of *S.cerevisiae *and 2,885,670 bp of the other fungi. After processing the 2,383,802 sequence pairs (generated from the target regions using a dual sliding window) by Cofolga2 and the SVM we trained, we obtained 2,807 pairwise alignments which have SVM probabilities ≥ 0.9. The *S. cerevisiae *sequences of the obtained pairwise alignments were clustered into 'ncRNA candidate regions' by a single linkage clustering, where overlapped or neighboring sequences are clustered. The ncRNA search in yeast (with a PC cluster consisting of thirteen Pentium4 PCs) took approximately twenty days. The obtained candidate regions are summarized in Table 2. As shown in the table, we found ncRNA candidates at 714 intergenic regions, 1,311 protein-coding regions, and twenty known ncRNA regions in the *S. cerevisiae *genome. Based on the total number of alignments processed by Cofolga2 and the false positive rate (0.06%) obtained in the benchmark, we estimated the number of false positive alignments = 1430, leading to (the estimated number of false positive alignments)/(the number of alignments predicted as ncRNA) = 51%. This value is almost same with the corresponding value obtained in the human ncRNA finding by CMfinder [24].

**Table 2:** Summary of the predicted *S. cerevisiae *ncRNA candidates

		*S. cerevisiae*		
*P*_cutoff_	organism	int	orf	rna
0.90	*S. mikatae*	252 (27,760)	356 (40,529)	10 (786)
	*S. kudriavzevii*	247 (28262)	423 (49,395)	9 (692)
	*S. bayanus*	116 (12,230)	221 (26,143)	3 (279)
	*S. castellii*	105 (12,537)	260 (30,009)	0 (0)
	*S. kluyveri*	184 (18,904)	315 (36,471)	9 (460)
	*S. paradoxus*	118 (12,493)	177 (22,219)	2 (200)
	all	714 (102,652)	1,311 (197,116)	20 (2,267)

0.95	*S. mikatae*	83 (10,234)	108 (14,190)	3 (350)
	*S. kudriavzevii*	76 (9,862)	121 (15,905)	2 (256)
	*S. bayanus*	37 (4,692)	63 (8,109)	0 (0)
	*S. castellii*	32 (3,886)	79 (10,532)	0 (0)
	*S. kluyveri*	55 (6,566)	88 (11,564)	2 (210)
	*S. paradoxus*	30 (3,497)	47 (6,286)	1 (150)
	all	253 (37,128)	435 (64,974)	7 (966)

The 'int', 'orf', and 'rna' columns indicate the number of ncRNA candidate regions for the *S. cerevisiae *intergenic, orf-coding, and known RNA sequences, respectively. In parenthesis, their total length (nt) is shown. The 'organism' column indicates the counterpart of each genome comparison. The 'all' rows are the summary for all genome comparisons after eliminating positional overlaps. *P*_cutoff _is a cutoff value for the SVM classification probability. In this table, the results for *P*_cutoff _= 0.9 and *P*_cutoff _= 0.95 are shown.

**Figure 9:**
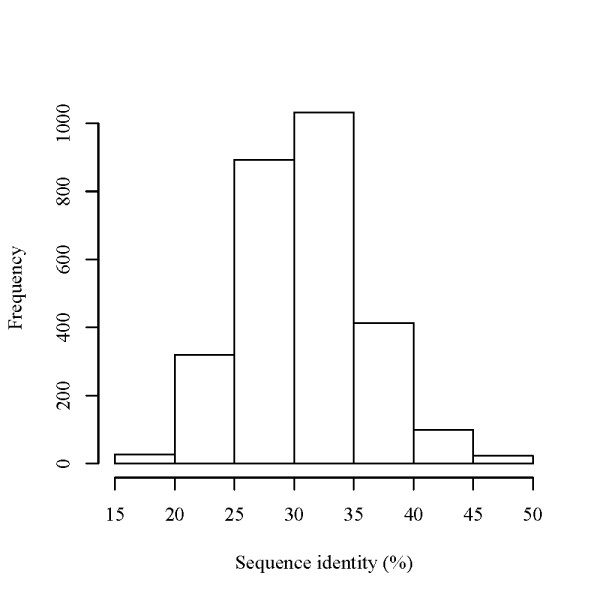
**Sequence identity distribution for the predicted ncRNA candidates**. The sequence identity distribution of the alignments with a significant SVM classification probability (≥ 0.9).

In the present predictions, we obtained 53 intergenic regions, 43 protein-coding regions, and 12 known ncRNA regions as ncRNA candidates (Table 3), which overlap at least one of the previous RNAz and QRNA predictions; where we classified a candidate as an "overlapped" region if ≥ 10% of the nucleotides of the candidate overlaps an RNAz or QRNA prediction. Relatively small overlaps between our ncRNA candidates and those by RNAz and QRNA are not surprising because our method does not require sequence conservation of ncRNA candidates while RNAz and QRNA directly utilize the sequence similarity between ncRNA candidates. For example, the lowest sequence identity in the alignments containing our ncRNA candidates was 15% (Figure 9).

**Table 3:** Overlaps between the predicted ncRNA candidates and the previsous predictions

region	RNAz	QRNA	Cofolga
intergenic	39 (5,941)	24 (3,556)	657 (94,705)
coding	45 (6,904)	0 (0)	1,266 (191,052)
known RNAs	11 (1,378)	12 (1,328)	6 (639)

The 'RNAz' and 'QRNA' columns indicate the number of the present *S. cerevisiae *ncRNA candidates overlapping with those by RNAz [49] and QRNA [4], respectively. In parenthesis, the total length (nt) for each region is mentioned. When ≥ 10% of the nucleotides of a ncRNA candidate overlaps the RNAz or QRNA prediction, we included the ncRNA candidate in the overlapped ncRNA candidates. The 'Cofolga' column indicates the ncRNA candidates predicted by Cofolga2 alone, i.e. novel ncRNA candidates obtained in the present study. Since the ncRNA candidate list of QRNA (Table S1 of [4]) does not contain strand information, we took only positional overlap into account when we examine the overlaps between the present ncRNA candidates and those by QRNA.

**Figure 10:**
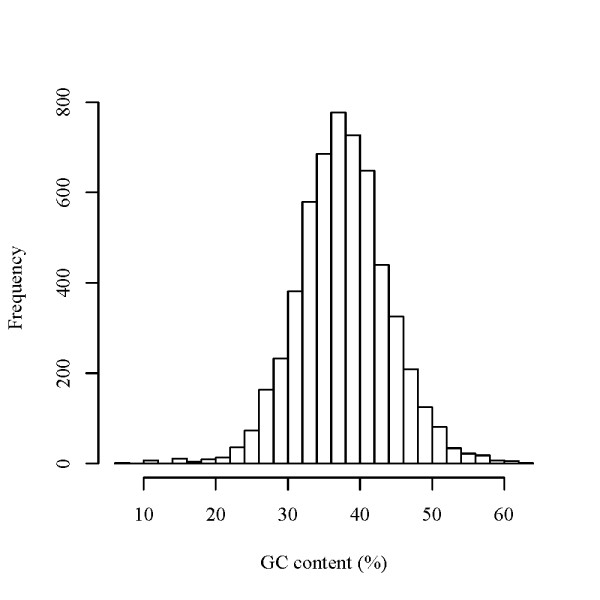
**Histogram of the GC content for the yeast ncRNA candidates**. The GC content of the *S. cerevisiae *ncRNA candidates distributed around the average GC content (approximately 37%) of *S. cerevisiae *auto chromosomes.

Figure 10 shows the histogram of the GC content for all ncRNA candidates we predicted. In the previous comparative ncRNA predictions [5] in which RNAz and EvoFold have been used to predict human ncRNAs, it was found that there are biases in the base composition distribution of the predicted ncRNA sequences, i.e. RNAz favors GC-rich sequences, while Evofold tends to predict AU-rich ones as ncRNA candidates [49]. As indicated in Figure 10, our prediction result is not biased in GC content (the average GC content of whole DNA sequences of *S. cerevisiae *auto chromosomes is approximately 37%).

The target regions we obtained includes 82 known ncRNAs. Of these, eighteen loci were included in the alignments with *P*_*SVM *_≥ 0.9. The detail of the predicted known ncRNAs is summarized in Additional File 3. An estimated sensitivity for the ncRNA prediction calculated based on this observation is approximately 22%. It is noteworthy that Cofolga2 correctly predicted the strand of fifteen known ncRNAs, i.e. correct strand was assigned to approximately 83% of the eighteen known ncRNAs. In these strand predictions, we adopted the strand with the highest *P*_*SVM *_when *P*_*SVM *_≥ 0.9 was assigned to the both strands.

Table 4 shows how many ncRNA candidates overlap the loci of the experimentally determined transcripts. In the table, experimental data taken from tilling array [50-52] and cDNA [53] are included. Here we found that the genomic positions of the results by David *et al*. and those by Miura *et al*. significantly (≥ 50% of the nucleotides of a candidate) overlap 112 and 69 ncRNA candidates of the present study, respectively, while our results show fewer overlaps with the transcripts by Davis *et al*. and Samanta *et al*.. Consequently, it turned out that 176 intergenic ncRNA candidates (this corresponding to approximately 25% of our intergenic candidates of *S. cerevisiae*) have at least one experimental support for their expression. By comparing the genomic positions of our results and the annotation (orf_coding.fasta) of SGD, we found that 95 and 71 intergenic candidates are located within 120 bp from the 5'-end and 3'-end of a CDS, respectively. In addition, eight intergenic candidates (SC000040I, SC000063I, SC000083I, SC000157I, SC000233I, SC000331I, SC000485I, SC000531I) are found at within 120 bp from the 5'- and 3'-ends of two CDSs, i.e. these eight candidates are sandwiched by two protein-coding genes. It is noted that the 5' and 3' ends of our ncRNA candidates can have an ambiguity of a few tens of nucleotides due to the gapped 5' and/or 3' edges of the pairwise alignments.

**Table 4:** Overlaps between the predicted ncRNA candidates and experimentally determined transcripts

region	David *et al*.	Davis *et al*.	Miura *et al*.	Samanta *et al*.	all
intergenic	112 (16,516)	37 (5,370)	69 (9,938)	2 (239)	176 (25,682)
coding	693 (109,508)	31 (4,454)	210 (32,721)	0 (0)	738 (116,286)
known RNA	7 (939)	8 (958)	5 (887)	0 (0)	12 (1,577)

The number of the predicted ncRNA candidates which overlap the loci of experimentally determined transcripts in literature. In parenthesis, the total length (nt) for each region is mentioned. Since the transcript list provided by Davis *et al*. (Supporting Table 1 in [51]) does not contain strand information, we took only positional overlap into account when we examine the overlaps between the present ncRNA candidates and the transcripts reported by Davis *et al*..

Recently, the ncRNAs found in protein-coding regions have been reported. In our prediction, we obtained more than one thousand ncRNA candidates in protein-coding regions. In these, 628 candidates are predicted at sense strand, and 684 candidates were predicted at antisense strand. One ncRNA candidate (SC000407F) simultaneously overlaps two protein-coding genes as sense and antisense since these two protein-coding genes overlap each other.

The detail of our prediction results and annotations can be browsed at our web server [40] in which the prediction results are retrieved through MySQL queries.

### ncRNA candidates conserved among multiple sequences

**Figure 11:**
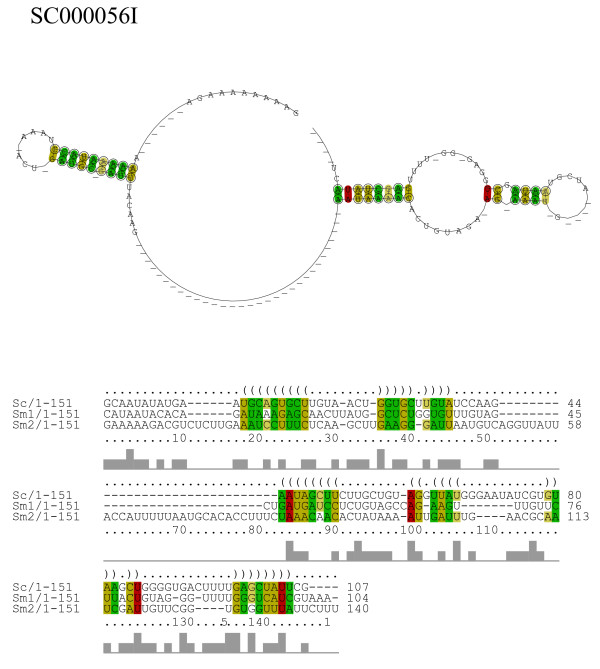
**Consensus structure and alignment of ncRNA candidate SC000056I**. This alignment is composed of a *S. cerevisiae *sequence (chr3/39,052–39,158 bp/+) and two *S. mikatae *sequences (c2601/5,712–5,861 bp/+ and c2601/6162–6311 bp/+, denoted by Sm1 and Sm2, respectively). The averege pair sequence identity of this alignment is 31%.

**Figure 12:**
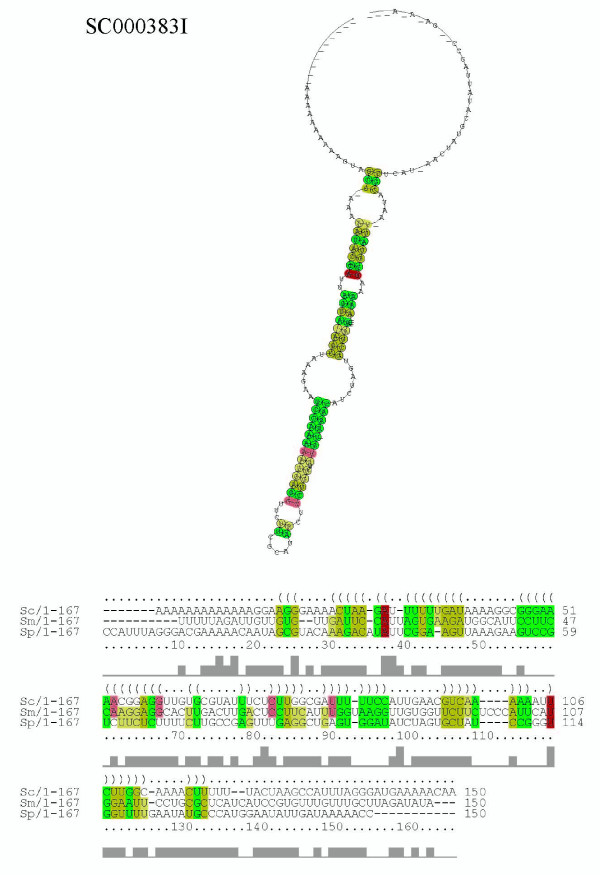
**Consensus structure and alignment of ncRNA candidate SC000383I**. This alignment is composed of a *S. cerevisiae *sequence (chr10/550,008–550,157 bp/-), a *S. mikatae *sequence (c2712/2,245–2,394 bp/-), and a *S. paradoxus *sequence (c303/5,880–6,029 bp/-). The averege pair sequence identity of this alignment is 28%.

By manually inspecting our prediction results, we found four intriguing examples containing conserved secondary structures across multiple species/sequences which have characteristic secondary structures in spite of their low average sequence identities. Figure 11 shows the alignment and structure of an intergenic *S. cerevisiae *sequence and two paralogous sequences of *S. mikatae *taken from the ncRNA candidate SC000056I. Since genomic separation between these two *S. mikatae *sequences are small (302 bp), these two *S. mikatae *sequences are a possible ncRNA cluster. Figure 12 shows the *S. cerevisiae *sequence of an intergenic ncRNA candidate (SC000383I) which was found at 39,052 bp to 39,158 bp of chromosome 3 and aligned with the sequences of *S. mikatae *and *S. paradoxus*. As can be seen from figures 11 and 12, these ncRNA candidates reveal characteristic consensus secondary structures in spite of their low average pair sequence identities (31%id for SC000056I and 28%id for SC000383I).

**Figure 13:**
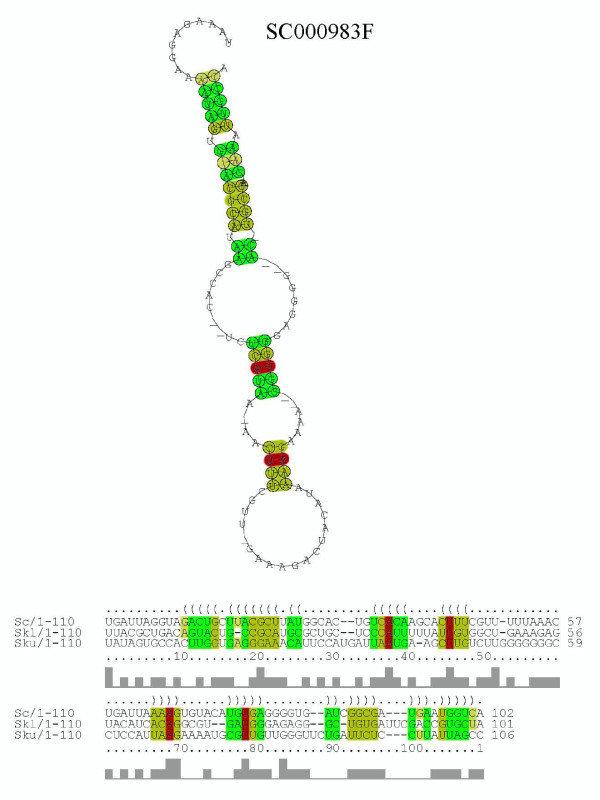
**Consensus structure and alignment found in ncRNA candidate SC000983F**. This alignment is composed of a *S. cerevisiae *sequence (chr13/663,965–664,066 bp/-), a *S. kluyveri *sequence (c2074/4,854–4,954 bp/-), and a *S. kudriavzevii *sequence (c2037/10,962–11,067 bp/-). The averege pair sequence identity of this alignment is 27%.

The ncRNA candidate SC000983F is one of the longest regions predicted in the present study. This candidate contains a consensus secondary structure motif shared by three species (Figure 13). In addition to the structure shared by three species, ncRNA candidate SC000983F contains a relatively long (300 bp) secondary structure (Additional File 4) conserved between two species (*S. cerevisiae *and *S. kudriavzevii*). It is noteworthy that almost all sequences contained in SC000983F are antisense sequences. The sequences of *S. cerevisiae *are antisense sequences of a gene *CLN1 *coding G1 cyclin which is involved in regulation of the cell cycle, and the sequences of *S. kluyveri *and *S. kudriavzevii *are antisense sequences of predicted ORFs according to the SGD annotation. These results imply that this ncRNA candidate is a functional antisense ncRNA with characteristic secondary structures.

**Figure 14:**
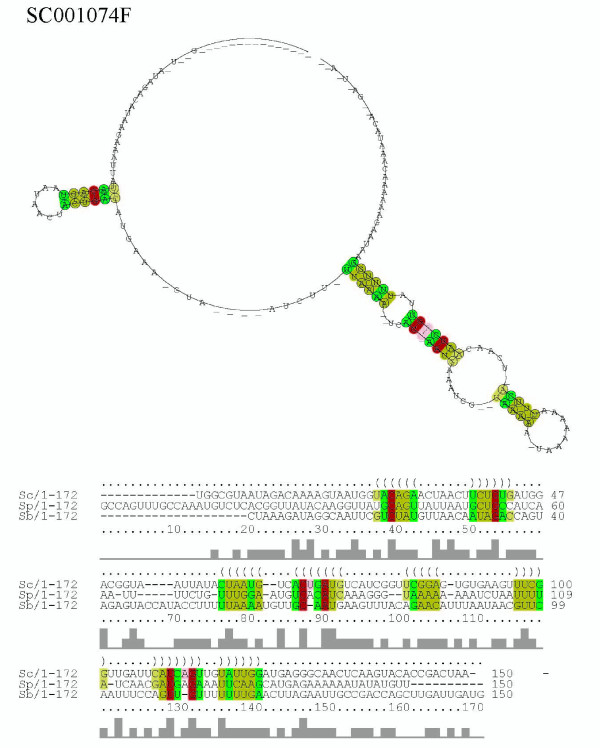
**Consensus structure and alignment of ncRNA candidate SC001074F**. This alignment is composed of a *S. cerevisiae *sequence (chr14/474,642–474,791 bp/+), a *S. paradoxus *sequence (c250/6,937–7,086 bp/+), and a *S. bayanus *sequence (c627/32,967–33,116 bp/+). The averege pair sequence identity of this alignment is 34%.

The *S. cerevisiae *sequence of ncRNA candidate SC01074F is located at the sense strand of *PMS1*, a verified ORF coding an ATP-binding protein. The sequences of *S. paradoxus *and *S. bayanus*, which overlap the predicted ORFs of each genome according to the SGD annotation, are structurally aligned to the *S. cerevisiae *sense sequence in SC01074F (Figure 14). These sequences are new candidates of functional RNA secondary structure within a coding region such as the localization elements of *ASH1 *which do not show sequence conservation but harbor conserved RNA secondary structure [54]. The multiple alignments for SC000056I, SC000383I, SC000983F, and SC001074F were constructed by manual operation (including partial re-alignment by a progressive alignment with Cofolga2) based on the pairwise alignments by Cofolga2. Since the progressive multiple alignment using Cofolga2 has not fully tested yet, we didn't benchmark it in this paper. The figures of consensus secondary structure and alignment were drawn by processing the multiple alignments at RNAalifold web server [34].

The examples described in this section (SC000056I, SC000383I, SC000983F, and SC01074F) have at least one experimental evidence for their expression according to the tilling array/cDNA data in literature [50-53].

## Conclusion

As can be known from a number of recent papers describing various structural RNA sequence alignment programs, it is a difficult problem to find a good RNA alignment with low sequence conservation. In the present study, we developed a new efficient GA for constructing structural RNA pairwise alignment with a new objective function and random alignment generation algorithm. The new GA is accurate and efficient in both time and memory usage, hence we applied it to the comparative ncRNA discovery between *S. cerevisiae *and related species using a SVM trained with the sequences and alignments taken from BRAliBase 2.1. As a result, we successfully obtained ncRNA candidates located at 714 intergenic regions and 1,311 protein-coding regions including antisense sequences, > 92% of which is novel candidates since they show no overlaps with the genomic positions of the previous predictions and known ncRNAs. Indeed, our approach is not suitable for identifying all ncRNA sequences embedded in a genome, it gives a valuable tool complementary to the sequence-alignment-based ncRNA finders such as RNAz and QRNA, since the present method often found the ncRNA candidates which cannot be found by such sequence-alignment-based ncRNA finders. The results of the present study indicate that still a number of structured RNA transcripts with significant structural and evolutional signals is hidden in genomic sequences, and further exploration for novel ncRNAs using computational methods is inevitable to unveil the RNomics of genomes.

## Availability and requirements

Non-profit, academic users can download and use the executable files at the Cofolga2 website [40].

## Supplementary Material

Additional File 1 **The file names of the RNA sequence pairs used in the GA parameter determination and memory usage test**. The top fourteen files were used for the GA parameter determination and convergence test. The other one (denoted by *) was used only for examining the maximum memory usage. Click here for file

Additional File 2 **Initial random number dependence of the benchmark result**. The benchmark results for the BRAliBase2.1 k2-dataset with five different initial random numbers. The results denoted by *R *= 12345 are same with those of Figure 2, since it is a default value. In addition, this figure includes the result obtained with a larger population size (= 100) with *R *= 12345. Click here for file

Additional File 3 **Known ncRNAs predicted in the present comparative genomics**. The 'ID' column indicates the index assigned for the predicted ncRNA candidates in the present study. The 'Same/Diff' column shows whether the strand of known RNAs are correctly predicted or not, where 'Same' and 'Diff' indicate "strand is correctly predicted" and "not correctly predicted", respectively. For snR63 and NME1, both strands are predicted as ncRNA candidates. The 'Chr.', 'begin', 'end', and 'strand' columns give the genomic positions and strand of the ncRNA candidates. The 'RNA name', 'SGDID', and 'SGD annotation' columns correspond to ncRNA gene names, IDs, and annotations given in SGD, respectively. Click here for file

Additional File 4 **Consensus structure and alignment of a sequence pair taken from ncRNA candidate SC000983F**. This alignment is composed of a sequence of *S. cerevisiae *(chr13/663,564–663,863 bp/-) and a sequence of *S. kudriavzevii *(c2037/10,520–10,819 bp/-). The sequence identity of this alignment is 32%. Click here for file
