# Supplementary material for: An efficient genetic algorithm for structural RNA pairwise alignment and its application to non-coding RNA discovery in yeast
Source: BMC Bioinformatics. 2008 Dec 5;9:521. doi: 10.1186/1471-2105-9-521 (PMC2630964; doi:10.1186/1471-2105-9-521)
Supplement: Additional File 1 — The file names of the RNA sequence pairs used in the GA parameter determination and memory usage test. The top fourteen files were used for the GA parameter determination and convergence test. The other one (denoted by *) was used only for examining the maximum memory usage. [file 1471-2105-9-521-S1.pdf]

Additional File 1 - The file names of the RNA sequence pairs used in the GA parameter determination and memory usage test

The top fourteen files were used for the GA parameter determination and convergence test. The other one (denoted by *) was used only for examining the maximum memory usage.

file names in the k2 dataset of BRAliBase 2.1
tRNA.apsi-19.sci-117.no-1.raw.fa
tRNA.apsi-19.sci-135.no-1.raw.fa
tRNA.apsi-40.sci-134.no-1.raw.fa
tRNA.apsi-40.sci-87.no-1.raw.fa
tRNA.apsi-70.sci-106.no-1.raw.fa
tRNA.apsi-70.sci-120.no-1.raw.fa
5S_rRNA.apsi-37.sci-107.no-1.raw.fa
5S_rRNA.apsi-37.sci-89.no-1.raw.fa
5S_rRNA.apsi-70.sci-129.no-1.raw.fa
5S_rRNA.apsi-70.sci-68.no-1.raw.fa
SRP_euk_arch.apsi-28.sci-70.no-1.raw.fa
SRP_euk_arch.apsi-28.sci-90.no-1.raw.fa
SRP_euk_arch.apsi-69.sci-103.no-1.raw.fa
SRP_euk_arch.apsi-70.sci-90.no-1.raw.fa
SRP_euk_arch.apsi-62.sci-75.no-1.raw.fa*
