# Supplementary material for: An efficient genetic algorithm for structural RNA pairwise alignment and its application to non-coding RNA discovery in yeast
Source: BMC Bioinformatics. 2008 Dec 5;9:521. doi: 10.1186/1471-2105-9-521 (PMC2630964; doi:10.1186/1471-2105-9-521)
Supplement: Additional File 2 — Initial random number dependence of the benchmark result. The benchmark results for the BRAliBase2.1 k2-dataset with five different initial random numbers. The results denoted by R = 12345 are same with those of Figure 2, since it is a default value. In addition, this figure includes the result obtained with a larger population size (= 100) with R = 12345. [file 1471-2105-9-521-S2.pdf]

Additional File 2 - Initial random number dependence of the benchmark result

The benchmark results for the BRAliBase2.1 k2-dataset with five different initial random numbers. The results denoted by $R = 12345$ are same with those of Figure 2, since it is a default value. In addition, this figure includes the result obtained with a larger population size (= 100) with $R = 12345$.

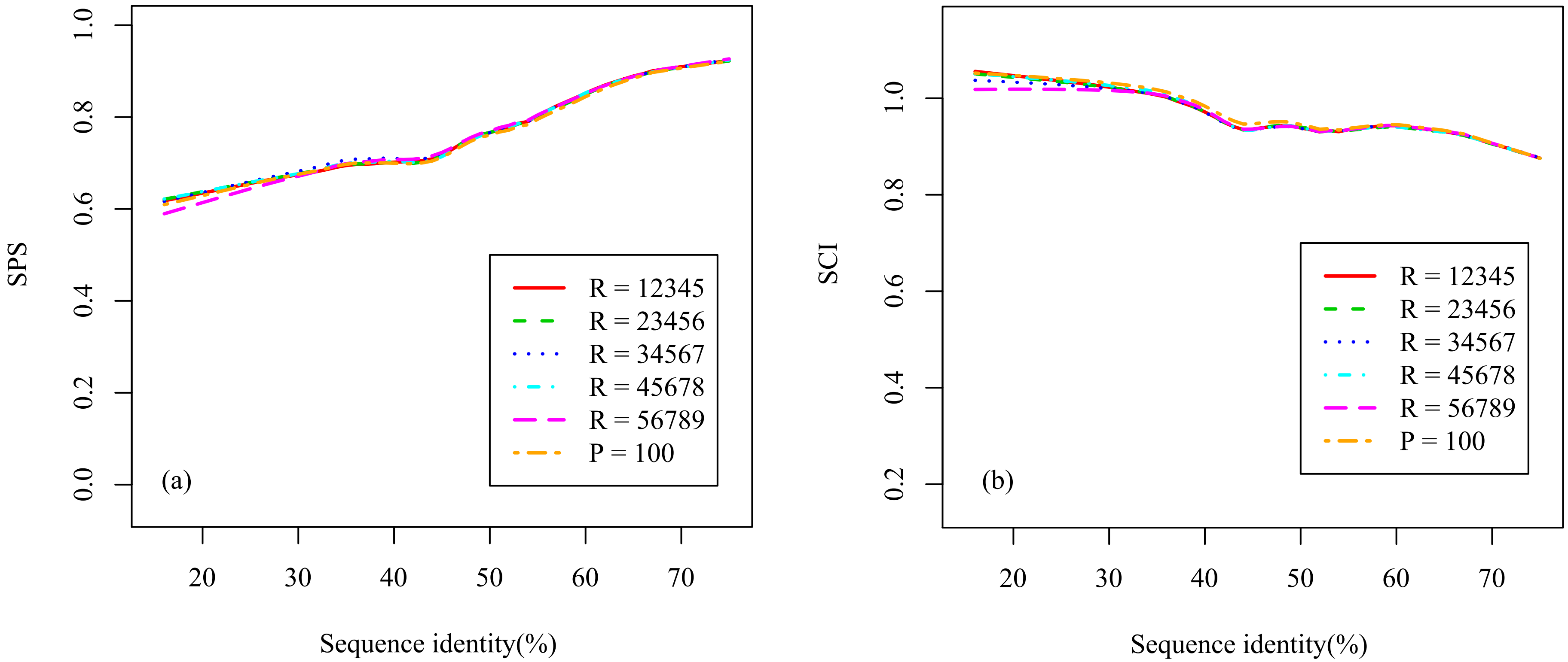

SPS
1.0
0.8
0.6
0.4
0.2
0.0
20
30
40
50
60
70
Sequence identity(%)
R = 12345
R = 23456
R = 34567
R = 45678
R = 56789
P = 100
(a)
SCI
1.0
0.8
0.6
0.4
0.2
(b)
