# Supplementary material for: An efficient genetic algorithm for structural RNA pairwise alignment and its application to non-coding RNA discovery in yeast
Source: BMC Bioinformatics. 2008 Dec 5;9:521. doi: 10.1186/1471-2105-9-521 (PMC2630964; doi:10.1186/1471-2105-9-521)
Supplement: Additional File 3 — Known ncRNAs predicted in the present comparative genomics. The 'ID' column indicates the index assigned for the predicted ncRNA candidates in the present study. The 'Same/Diff' column shows whether the strand of known RNAs are correctly predicted or not, where 'Same' and 'Diff' indicate "strand is correctly predicted" and "not correctly predicted", respectively. For snR63 and NME1, both strands are predicted as ncRNA candidates. The 'Chr.', 'begin', 'end', and 'strand' columns give the genomic positions and strand of the ncRNA candidates. The 'RNA name', 'SGDID', and 'SGD annotation' columns correspond to ncRNA gene names, IDs, and annotations given in SGD, respectively. [file 1471-2105-9-521-S3.pdf]

Additional File 3 - Known ncRNAs predicted in the present comparative genomics

The 'ID' column indicates the index assigned for the predicted ncRNA candidates in the present study. The 'Same/Diff' column shows whether the strand of known RNAs are correctly predicted or not, where 'Same' and 'Diff' indicate "strand is correctly predicted" and "not correctly predicted", respectively. For snR63 and NME1, both strands are predicted as ncRNA candidates. The 'Chr.', 'begin', 'end', and 'strand' columns give the genomic positions and strand of the ncRNA candidates. The 'RNA name', 'SGDID', and 'SGD annotation' columns correspond to ncRNA gene names, IDs, and annotations given in SGD, respectively.

ID	Same/Diff	Chr.	begin	end	strand	RNA name	SGDID	SGD annotation
SC000001R	Same	3	107504	107712	-	snR43	S000006503	"H/ACA snoRNA; predicted to guide pseudouridylation of large subunit (LSU) rRNA at position U966"
SC000002R	Diff	4	323217	323471	-	snR63	S000006454	"C/D snoRNA; guides 2'-O-methylation of large subunit (LSU) rRNA at position A2256"
SC000003R	Same	4	323217	323471	-	snR63	S000006454	"C/D snoRNA; guides 2'-O-methylation of large subunit (LSU) rRNA at position A2256"
SC000004R	Same	4	1492469	1493018	-	snR84	S000028466	"H/ACA snoRNA; guides pseudouridylation of large subunit (LSU) rRNA at position U2266"
SC000005R	Same	5	117667	118035	-	RPR1	S000006490	"RNA component of nuclear RNase P which cleaves tRNA precursors"
SC000006R	Same	5	431125	431216	-	snR52	S000006443	"C/D snoRNA; guides 2'-O-methylation of large subunit (LSU) rRNA at position U2921 and small subunit (SSU) rRNA at position A420"
SC000007R	Same	8	133019	133100	-	tS(AGA)H	S000006726	"tRNA-Ser"
SC000008R	Same	8	134314	134385	+	tQ(UUG)H	S000006698	"tRNA-Gln"
SC000009R	Same	11	282829	282923	+	snR38	S000007301	"C/D snoRNA; guides 2'-O-methylation of large subunit (LSU) rRNA at position G2815"
SC000010R	Same	12	167943	168024	-	tS(AGA)L	S000006728	"tRNA-Ser"
SC000011R	Diff	12	198785	199390	+	snR30	S000007497	"H/ACA snoRNA; essential gene required for cleavage of 35S primary rRNA transcript to release the precursor to the 18S rRNA"
SC000012R	Same	12	448651	448722	-	tQ(UUG)L	S000006699	"tRNA-Gln"
SC000013R	Same	13	259158	259239	-	tS(AGA)M	S000006729	"tRNA-Ser"
SC000014R	Same	13	420587	420660	-	tV(AAC)M2	S000006764	"tRNA-Val"
SC000015R	Same	13	652274	652531	+	snR11	S000007293	"H/ACA snoRNA; guides pseudouridylation of large subunit (LSU) rRNA at position U2416"
SC000016R	Same	14	585588	585927	+	NME1	S000007436	"RNA component of RNase MRP which cleaves pre-rRNA"
SC000017R	Diff	14	585588	585927	+	NME1	S000007436	"RNA component of RNase MRP which cleaves pre-rRNA"
SC000018R	Diff	15	832331	832520	+	snR8	S000006497	"H/ACA snoRNA; guides pseudouridylation of large subunit (LSU) rRNA at positions U960 and U986"
SC000019R	Same	15	842402	842605	+	snR5	S000007291	"H/ACA snoRNA; guides pseudouridylation of large subunit (LSU) rRNA at positions U1004 and U1124"
SC000020R	Same	16	281055	281372	-	snR17b	S000007441	intron sequence removed snoRNA "snoRNA U3; part of small (ribosomal) subunit (SSU) processosome"
