# Supplementary material for: An efficient genetic algorithm for structural RNA pairwise alignment and its application to non-coding RNA discovery in yeast
Source: BMC Bioinformatics. 2008 Dec 5;9:521. doi: 10.1186/1471-2105-9-521 (PMC2630964; doi:10.1186/1471-2105-9-521)
Supplement: Additional File 4 — Consensus structure and alignment of a sequence pair taken from ncRNA candidate SC000983F. This alignment is composed of a sequence of S. cerevisiae (chr13/663,564–663,863 bp/-) and a sequence of S. kudriavzevii (c2037/10,520–10,819 bp/-). The sequence identity of this alignment is 32%. [file 1471-2105-9-521-S4.pdf]

Additional File 4 - Consensus structure and alignment of a sequence pair taken from ncRNA candidate SC000983F

This alignment is composed of a sequence of *S. cerevisiae* (chr13/663,564-663,863bp/-) and a sequence of *S. kudriavzevii* (c2037/10,520-10,819bp/-). The sequence identity of this alignment is 32%.

SC000983F

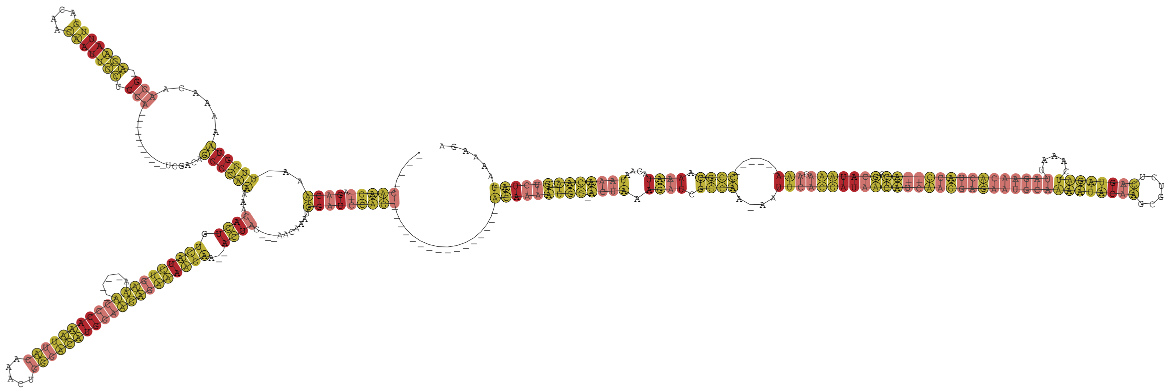

```
            ....(((((.((((...((((((......(((.(((((((....))))))).))).....
Sc/1-328    UGAUGAAG-AGGCAUU-UUGGUCAAAGUAACG-AGAAUUGACAGCAAUUUUUCUGGUUAG 57
Sku/1-328   ----GCUGCUGACCAAAUUGGUACAUACUUGGUGUGAUUGGUGAUGAUUGCUCCA----- 51
            .........10........20........30........40........50.........

            ............))))))...((((.(((((((((.....(((((((((((.....))))
Sc/1-328    GUCUAUUGUAGAGGCCAGUUGCAGUUUCUUUUGUAA----UUCCAAGUUAUAAUCUUUUA 113
Sku/1-328   ------UGGUCCUGCUGAAAACACUGUUAUCUGAUAAAGAACUCAUAUUUCUCAUUGGGG 105
            .........70........80........90........100.......110........

            ))))))))))))))))...))))..........)))))))))................((
Sc/1-328    CUUGUAAUAUGAAAGGU--ACUAU---ACCAUAUUGCUCCUU----------------AU 152
Sku/1-328   UAUGGCUGUGAGAUGAAUAAGUGGGCGAAGGAAUGGAUGUAGUGUGUGAAUUUUCAAAGC 165
            .........130.......140.......150.......160.......170........

            (((((((.((((.((((.((((....((((((((((((((((((((((((((((((((((
Sc/1-328    AAACUUCGACUAAAGAUGGGGGAGAAUUGACUAUGCCAUUGAUGAUAAUGCUGAAUACCU 212
Sku/1-328   AACAUGG-UGUGGAUAUCGUCAG-UUUUCUUGAUAAGUUCAAGUUGGAUGCAAUUUGCAA 223
            .........190.......200.......210.......220.......230........

            ......)))))))).....)))))))))))))))).))))))).)))......)))).))
Sc/1-328    GCGUUUGAGUGUUUGAGUUUAUAUCAUUACCAAUGGGCAUAGAGAGAAAAGGGCCUUGAU 272
Sku/1-328   UUGUCUUUGUAGAUCCAAUUGGUAUCCUCUU--ACUUGAUCAAUGAA------UGGCAAA 275
            .........250.......260.......270.......280.......290........

            ))....)))))))...))))))......
Sc/1-328    CUUGAUUAGUGAAUUUGUUUAUUAUAGA 300
Sku/1-328   AUACCAUUAACCG---UUCUGUAAACUU 300
            .........310.......320......
```
